# Growable Shunt as Novel Therapy in Children Requiring Aortopulmonary Shunt for Extended Time

**DOI:** 10.1016/j.atssr.2023.02.023

**Published:** 2023-03-08

**Authors:** Alexander P. Bruno, Daniel K. Ragheb, Dana R. Janssen, David P. Bichell

**Affiliations:** 1 Vanderbilt University School of Medicine, Nashville, Tennessee; 2 Department of Cardiology, Monroe Carell Jr Children’s Hospital, Vanderbilt University Medical Center, Nashville, Tennessee; 3 Department of Cardiac Surgery, Monroe Carell Jr Children’s Hospital, Vanderbilt University Medical Center, Nashville, Tennessee

## Abstract

We describe a 35-month-old girl with a complex congenital heart defect including double-inlet left ventricle who presented with an occluded ductal stent. We performed pulmonary artery unifocalization and central shunt placement to bridge to Fontan or as final palliation. Anticipating a prolonged role for the shunt, we used a novel approach to aortopulmonary shunt construction consisting of a stent-restricted shunt that allows extended shunt longevity through an ability to “grow” with the patient’s somatic growth. This technique demonstrates a safe way to dilate aortopulmonary shunts without repeated open surgical intervention.

The systemic-to-pulmonary shunt, as an interim source of pulmonary blood flow for single-ventricle and biventricular patients, bridges to subsequent palliative or reparative procedures for a duration of few to many months. The diameter of the shunt is the greatest effector of pulmonary flow and is critically important to patients, especially in the immediate postoperative period when cardiac output is limited. Smaller shunts are favorable in smaller patients to best balance systemic and pulmonary circulations and to maintain optimal end-organ perfusion. Shunt enlargement ideally accompanies somatic growth to maintain physiologic balance and to drive optimal pulmonary artery (PA) growth. For patients with PA hypoplasia or elevated pulmonary resistance that may require an aortopulmonary shunt for a protracted period, determining the ideal shunt diameter is further complicated by conflicting short- and long-term hemodynamic demands. In addition, a smaller, fixed-diameter shunt that does not have the ability to grow will inevitably become insufficient with somatic growth, leading to progressive cyanosis and requiring repeated operations to upsize. Thus, for patients requiring an aortopulmonary shunt for protracted periods, we have developed a method of shunt construction with a luminal diameter that is adjustable with growth without the need for repeated open surgical interventions.

We report the case of a 35-month-old girl with double-inlet left ventricle, dextrocardia, and pulmonary atresia with an occluded ductal stent placed in infancy in China, discontinuous and asymmetrically hypoplastic PAs, aortopulmonary collaterals, and severe cyanosis. As a bridge to suitable pulmonary vascular health for Fontan completion or as destination palliation, we elected to perform PA unifocalization and central shunt placement.

Anticipating a prolonged role for the aortopulmonary shunt, an intentionally oversized, 6-mm polytetrafluoroethylene (PTFE) conduit was constructed between the ascending aorta and right PA. At construction, the shunt was threaded through an expandable stent. The stent was crimped uniformly to externally constrict the shunt to limit the initial pulmonary blood flow from the arterial supply; anastomoses at shunt origin and insertion remained large (Figures 1, 2). The shunt continued to be restricted until oxygen saturation was in the 80s on 50% fraction of inspired oxygen, resulting in an effective shunt diameter of 4 mm.

The postoperative course included inotrope support for 24 hours; initial hypoxia with peripheral oxygen saturation of 65% improved on pulmonary hygiene, on room air, by postoperative day 3. Her postoperative course was not otherwise complicated, and she was discharged on postoperative day 7.

Three months later, she presented for scheduled catheterization for stent expansion, clinically indicated for desaturations on physical activity (75% at rest, 59% during activity). The aortopulmonary shunt was balloon dilated to 6 mm, leading to angiographic improvement and improved saturations (Figure 1). The patient also had mild narrowing of the left PA, which was dilated to 6 mm. The patient was discharged the next day.

During the ensuing 12 months, she reported increased activity level with oxygen saturation in the 80s. One year after dilation, the patient underwent catheterization that demonstrated mean PA pressure of 12 mm Hg, ventricular end-diastolic pressure of 3 mm Hg, and normal-sized, symmetric branch PAs. At the age of 4 years, she had a successful Fontan operation.

## Comment

We report a novel approach to aortopulmonary shunt construction using a stent-restricted PTFE shunt that allows extended shunt longevity through an ability to “grow” with the patient’s anatomy. This technique demonstrates a safe way to dilate aortopulmonary shunts without repeated open surgical intervention.

Several previous studies reported success with dilation of aortopulmonary shunts in infants. Gewillig and colleagues^1^ described dilating Laks-type shunts (vascular grafts anastomosed end-to-side to the PA and side-to-side to the anterior aorta) using stretch PTFE vascular grafts. They discussed the need to use the Laks-type shunt because of technical difficulties associated with percutaneous intervention for the modified Blalock-Taussig shunt. Penford and colleagues^2^ also reported success with stenting and overdilation of vascular PTFE shunts (ventriculopulmonary, central, and Blalock-Taussig-Thomas shunts) for shunt dilation due to desaturations, reduced graft flow on imaging, and graft endocarditis or blockages or to postpone staged surgery. They reported a significant increase in oxygen saturation after stent dilation with a mean increase of 13%. Other reports described percutaneous intervention for central and Blalock-Taussig-Thomas shunts, mostly for shunt stenosis or occlusion and mostly describing the insertion of stents within shunts.^3^, ^4^, ^5^, ^6^ The exGraft (PECA Labs), a PTFE conduit capable of balloon dilation to 150% original diameter, is in limited clinical use at present.^7^ Fixed anastomotic diameter, angle of origin or insertion, and size and angle of pre-shunt or post-shunt vessels may limit the effectiveness of shunt dilation in some of the described settings. Oversized anastomoses in an intentionally oversized shunt with midshunt restriction by external stent may permit a more uncomplicated, uniform dilation of the midshunt to match origin and insertion.

For patients requiring an aortopulmonary shunt for a protracted time, an oversized shunt, stent restricted externally at implantation, allows graded dilation to match demand over time, in keeping with somatic growth and relative resistances required to balance systemic and pulmonary circulations. We describe a successful application of the novel method of shunt construction, with interval dilation that improved pulmonary blood flow to match the patient’s changing hemodynamic demands.

**Figure 1:**
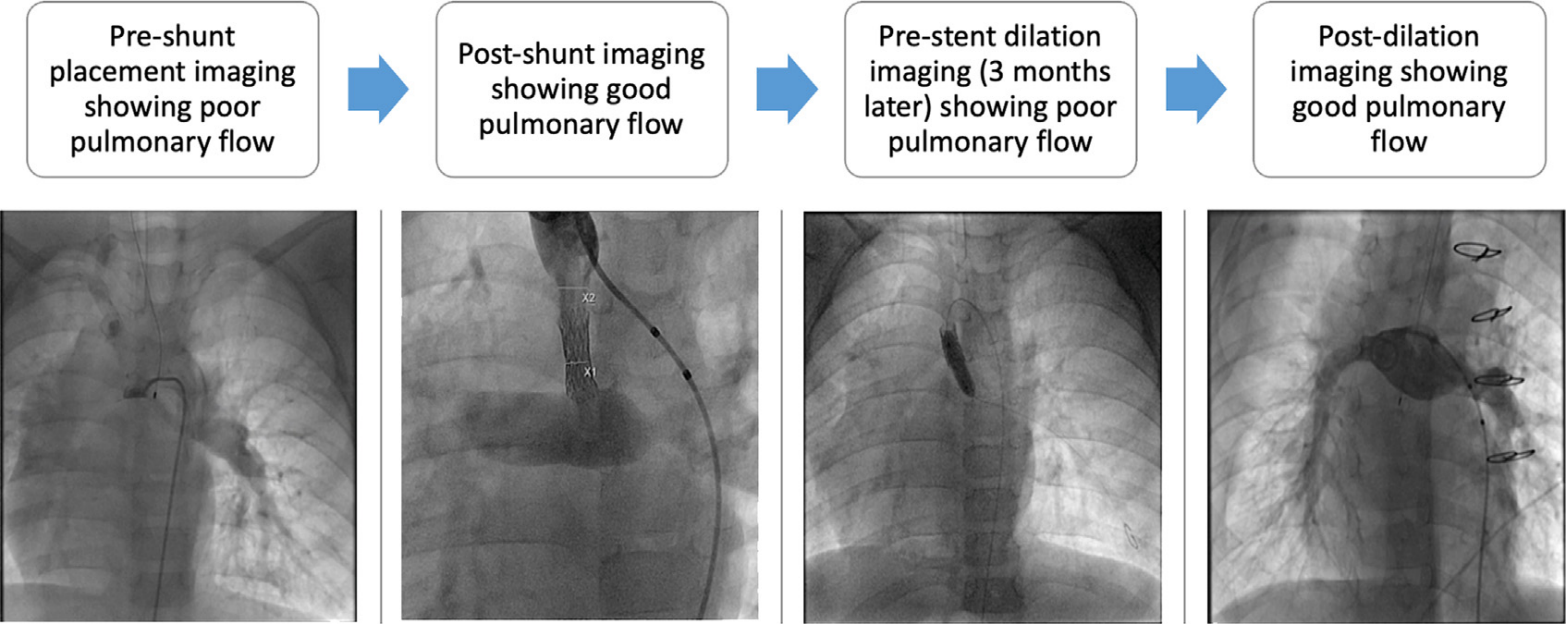
Series of catheter images showing patient’s course through stent placement, scheduled dilation, and 1-year follow-up.

**Figure 2:**
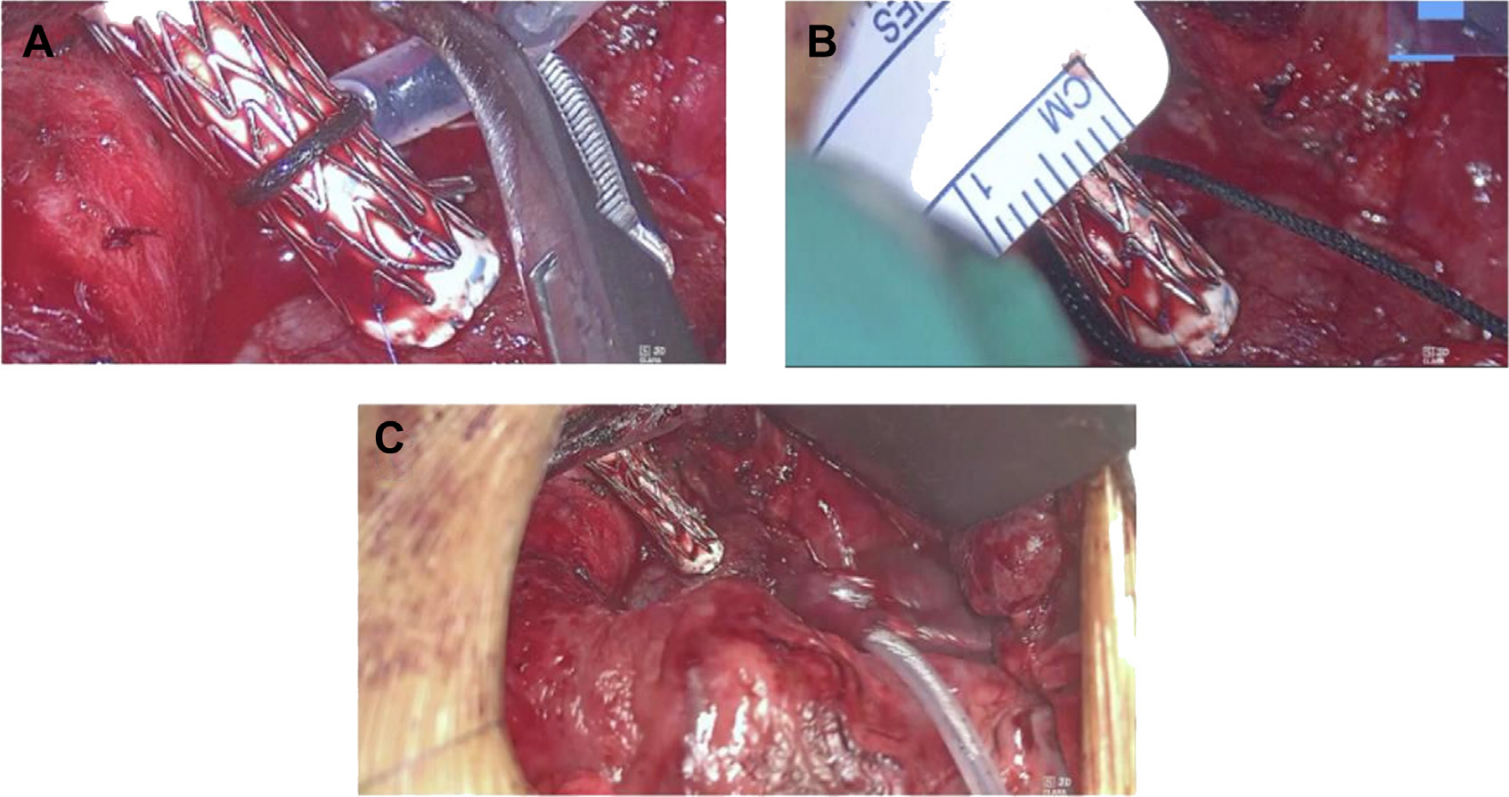
(A) Central shunt placement with external stent, about to be crimped. (B) Crimped stent restricting shunt to total diameter of 4 mm. (C) Fully crimped stent around downsized shunt in final placement at surgical completion.
